# Coordination of mitochondrial and lysosomal homeostasis mitigates inflammation and muscle atrophy during aging

**DOI:** 10.1111/acel.13583

**Published:** 2022-03-09

**Authors:** Andrea Irazoki, Marta Martinez‐Vicente, Pilar Aparicio, Cecilia Aris, Esmaeil Alibakhshi, Maria Rubio‐Valera, Juan Castellanos, Luis Lores, Manuel Palacín, Anna Gumà, Antonio Zorzano, David Sebastián

**Affiliations:** ^1^ Institute for Research in Biomedicine (IRB Barcelona) The Barcelona Institute of Science and Technology Barcelona Spain; ^2^ Departament de Bioquímica i Biomedicina Molecular Facultat de Biologia Universitat de Barcelona Barcelona Spain; ^3^ Centro de Investigación Biomédica en Red de Diabetes y Enfermedades Metabólicas Asociadas (CIBERDEM) Instituto de Salud Carlos III Barcelona Spain; ^4^ Neurodegenerative Diseases Research Group Vall d’Hebron Research Institute‐Center for Networked Biomedical Research on Neurodegenerative Diseases (CIBERNED) Barcelona Spain; ^5^ Department of Orthopedic Surgery and Traumatology Hospital General Parc Sanitari Sant Joan de Déu Sant Boi de Llobregat, Barcelona Spain; ^6^ Department of Family and Community Medicine Hospital General Parc Sanitari Sant Joan de Déu Sant Boi de Llobregat, Barcelona Spain; ^7^ Pneumology Department Hospital General Parc Sanitari Sant Joan de Déu Sant Boi de Llobregat, Barcelona Spain; ^8^ Physical Medicine and Rehabilitation Department Clinical Research Development Unite Baqyiatallah Hospital, Faculty of Medicine Baqyiatallah University of Medical Science Tehran Iran; ^9^ Quantitative MR Imaging and Spectroscopy Group Research Center for Molecular and Cellular Imaging Advanced Medical Technologies and Equipment Institute Tehran University of Medical Science Tehran Iran; ^10^ Hospital General Parc Sanitari Sant Joan de Déu Sant Boi de Llobregat, Barcelona Spain; ^11^ The Biomedical Research Centre Network for Epidemiology and Public Health (CIBERESP) Madrid Spain; ^12^ CIBER de Enfermedades Raras (CIBERER) Instituto de Salud Carlos III Madrid Spain; ^13^ Institute of Biomedicine of the University of Barcelona (IBUB) Barcelona Spain

**Keywords:** aging, inflammation, lysosome, mitochondria, mitophagy, muscle

## Abstract

Sarcopenia is one of the main factors contributing to the disability of aged people. Among the possible molecular determinants of sarcopenia, increasing evidences suggest that chronic inflammation contributes to its development. However, a key unresolved question is the nature of the factors that drive inflammation during aging and that participate in the development of sarcopenia. In this regard, mitochondrial dysfunction and alterations in mitophagy induce inflammatory responses in a wide range of cells and tissues. However, whether accumulation of damaged mitochondria (MIT) in muscle could trigger inflammation in the context of aging is still unknown. Here, we demonstrate that BCL2 interacting protein 3 (BNIP3) plays a key role in the control of mitochondrial and lysosomal homeostasis, and mitigates muscle inflammation and atrophy during aging. We show that muscle BNIP3 expression increases during aging in mice and in some humans. BNIP3 deficiency alters mitochondrial function, decreases mitophagic flux and, surprisingly, induces lysosomal dysfunction, leading to an upregulation of Toll‐like receptor 9 (TLR9)‐dependent inflammation and activation of the NLRP3 (nucleotide‐binding oligomerization domain (NOD)‐, leucine‐rich repeat (LRR)‐, and pyrin domain‐containing protein 3) inflammasome in muscle cells and mouse muscle. Importantly, downregulation of muscle BNIP3 in aged mice exacerbates inflammation and muscle atrophy, and high BNIP3 expression in aged human subjects associates with a low inflammatory profile, suggesting a protective role for BNIP3 against age‐induced muscle inflammation in mice and humans. Taken together, our data allow us to propose a new adaptive mechanism involving the mitophagy protein BNIP3, which links mitochondrial and lysosomal homeostasis with inflammation and is key to maintaining muscle health during aging.

## Graphical abstract

Chronic inflammation is associated with the development of sarcopenia. In our study, we demonstrate that coordination of mitochondrial and lysosomal function by BCL2 interacting protein 3 (BNIP3) is key to limiting muscle inflammation. Importantly, muscle BNIP3 increases during aging in mice and humans, and this constitutes an adaptive mechanism that confers resistance to aging‐induced inflammation and muscle atrophy.

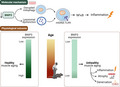

## 1 INTRODUCTION

Aging is associated with a progressive decline in muscle mass and function, a process known as sarcopenia. Sarcopenia is one of the main factors contributing to the disability of aged people, and therefore, extensive research is being done in order to decipher their molecular determinants. Aging is also associated with low‐grade chronic inflammation, also called inflammaging, which is involved in the pathogenesis of several disabling diseases that affect the elderly (Ferrucci & Fabbri, 2018). Epidemiological studies have found that inflammaging is a risk factor for the development of sarcopenia (Schaap et al., 2009; Soysal et al., 2016), and studies performed in mice indicate that blockage of NLRP3 (nucleotide‐binding oligomerization domain (NOD)‐, leucine‐rich repeat (LRR)‐, and pyrin domain‐containing protein 3) inflammasome attenuates its development (Sayed et al., 2019; Youm et al., 2013). However, the causes and precise mechanisms that trigger inflammation during aging and their implications in the development of sarcopenia are poorly understood.

Mitochondria exert key cellular functions and are essential for the maintenance of cellular health. Therefore, mitochondrial quality control mechanisms are necessary to prevent excessive damage to these organelles. In this regard, the removal of damaged mitochondria by mitochondrial autophagy, called mitophagy, has emerged as a crucial process in maintaining a healthy mitochondrial population within the cell (Onishi et al., 2021). Importantly, accumulation of damaged mitochondria and decreased mitophagy have been shown to occur during aging and to be associated with sarcopenia (Leduc‐Gaudet et al., 2021). Furthermore, alterations in mitophagy have been described to trigger inflammatory responses (Li et al., 2019; Rodriguez‐Nuevo et al., 2018; Sliter et al., 2018). However, whether altered mitophagy contributes to age‐induced inflammation in muscle, and whether this could be associated with sarcopenia are still unknown.

Mitochondria are a source of damage‐associated molecular patterns (DAMPs), and therefore, the accumulation of damaged mitochondria due to impaired mitophagy could result in the release of mitochondrial DAMPs (mtDAMPs), which in turn stimulates the activation of various inflammatory pathways. For instance, the mislocation of mitochondrial DNA (mtDNA), one of the most widely characterized mtDAMPs, triggers inflammation by distinct intracellular pathways. On the one hand, leakage of mtDNA to the cytosol activates the DNA sensor cyclic GMP–AMP synthase (cGAS), which promotes the stimulator of interferon genes–interferon regulatory factor 3 (STING–IRF3)‐dependent expression of type 1 interferon (IFN) response inflammatory genes (West et al., 2015). On the other hand, mtDNA has been reported to bind and activate Toll‐like receptor 9 (TLR9) in endosomes and lysosomes, inducing nuclear factor kappa B (NF‐κB) signaling and increasing the expression of other pro‐inflammatory cytokines (Oka et al., 2012).

BCL2 interacting protein 3 (BNIP3) is an atypical BH3 domain‐only member of the Bcl‐2 family of proteins (Chen et al., 1997). BNIP3 localizes in mitochondria, where it acts as a mitophagy receptor (Zhang & Ney, 2009). However, although extensively studied in cancer cell lines and cardiomyocytes (Zhang & Ney, 2009), the role of BNIP3 in mitochondrial homeostasis in skeletal muscle cells has not been addressed to date. Importantly, BNIP3 expression confers resistance to a variety of metabolic stresses. In fact, under hypoxic conditions, the stabilization of hypoxia‐inducible factor 1α (HIF1α) promotes the expression of BNIP3 in order to remove mitochondria, and the blockage of this pathway leads to cell death (Zhang et al., 2008). In skeletal muscle, exercise leads to an increase in BNIP3 expression, resulting in enhanced mitophagy (Lira et al., 2013), which is crucial for metabolic adaptation to exercise (Ehrlicher et al., 2020).

Interestingly, we have previously shown that aging and mitofusin 2 (Mfn2) deficiency in skeletal muscle lead to impaired mitophagy, mitochondrial dysfunction, and reactive oxygen species (ROS) production, which promote a BNIP3‐dependent compensatory mitophagic pathway whose inhibition causes further mitochondrial impairment and muscle atrophy (Sebastian et al., 2016). In the present study, we sought to further explore the role of BNIP3 in mitochondrial homeostasis in skeletal muscle during aging. We show that BNIP3 is crucial for the maintenance of mitochondrial health and lysosomal activity and that it confers resistance to aging‐induced muscle inflammation and atrophy.

## 2 RESULTS

### 2.1 BNIP3 is induced in aging, and it sustains mitochondrial function in muscle cells

**FIGURE 1:**
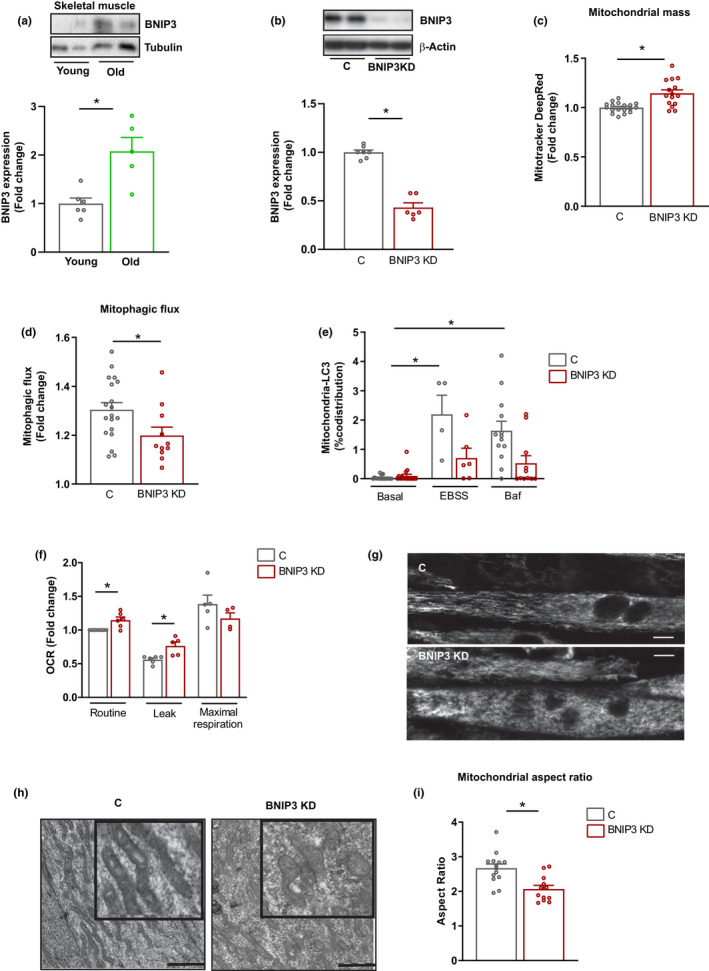
BCL2 interacting protein 3 (BNIP3) regulates mitophagy and mitochondrial health in skeletal muscle cells. (a) BNIP3 protein expression in gastrocnemius muscle from young (4–6 months) and old (22–24 months) wild‐type (WT) mice (*n* = 5–6 mice per group). (b) BNIP3 protein expression in control (C) and BNIP3 knockdown (BNIP3 KD) C2C12 myotubes (*n* = 6). (c) Mitochondrial mass measured by Mitotracker Deep Red fluorescence by flow cytometry in C and BNIP3 KD cells (*n* = 12). (d) Mitophagic flux in C and BNIP3 KD myotubes determined by Mitotracker Deep Red fluorescence in the absence or presence of 200 nM bafilomycin A for 16 h. Data show the fold change in fluorescence of bafilomycin A‐treated cells vs. nontreated cells (*n* = 12). (e) Quantification of co‐distribution between cyclooxygenase 1 (COX1) and microtubule‐associated protein 1A/1B‐light chain 3 (LC3) in C and BNIP3 KD myotubes under basal, starvation (Earle's Balanced Salt Solution (EBSS)) and bafilomycin A‐treated conditions (*n* = 5–20 myotubes). (f) Mitochondrial oxygen consumption in C and BNIP3 KD myotubes in routine, oligomycin‐treated (leak), and carbonyl cyanide *m*‐chlorophenylhydrazone (CCCP)‐treated (maximal) conditions (*n* = 6). (g) Representative live confocal microscopy images of C and BNIP3 KD myotubes incubated with Mitotracker Deep Red to visualize mitochondrial morphology (scale bar 10 µm); (h) Representative transmission electron microscopy (TEM) images of mitochondria from C and BNIP3 KD myotubes (scale bar 1 µm). (i) Quantification of mitochondrial morphology by aspect ratio measurement of mitochondria from TEM images of C and BNIP3 KD myotubes (*n* = 12–15 images and 20–25 mitochondria per image). Data are expressed as mean ± SE. *p < 0.05

We have previously shown that metabolic stress induced by Mfn2 repression triggers an increase in BNIP3 expression in skeletal muscle to preserve mitochondrial health (Sebastian et al., 2016). Here, we analyzed the muscle BNIP3 expression in young (4–6‐month‐old) and old (22–24‐month‐old) healthy C57BL/6J mice and found that aging was associated with a 2‐fold increase in the expression of this protein (Figure 1A).

To gain insight into the functional role of BNIP3, we transduced C2C12 myotubes with adenoviruses encoding for a short hairpin RNA (shRNA) against this protein. Gene and protein expression analyses showed a significant reduction of BNIP3 expression in silenced myotubes (BNIP3 knockdown (BNIP3 KD)) compared to controls (C) (Figure 1B; Figure S1A). Repression of BNIP3 increased mitochondrial mass, as indicated by higher Mitotracker Deep Red staining (Figure 1C), and enhanced the expression of the mitochondrial marker TIM44 (Figure S1B) and mtDNA copy number (Figure S1C). This increase in mitochondrial mass occurred in the presence of a reduced mitophagic flux, measured by Mitotracker Deep Red staining in basal and bafilomycin A‐treated conditions (Figure 1D; Figure S1D). To further characterize the mitophagy impairment in BNIP3 KD cells, co‐localization experiments between mitochondria and autophagic structures were performed. Analysis of the co‐distribution of mitochondria (detected by protein cytochrome c oxidase I (COX1)) and autophagosomes (detected by microtubule‐associated protein 1A/1B‐light chain 3 (LC3) II immunofluorescence) revealed a marked increase in control cells upon amino acid starvation (Earle's Balanced Salt Solution (EBSS)) and bafilomycin A treatment (Figure 1E; Figure S1E). However, this co‐distribution was significantly attenuated in BNIP3‐deficient cells, which indicates a reduced mitophagic flux (Figure 1E; Figure S1E). Furthermore, co‐localization of mitochondria with lysosomes (lysosomal‐associated membrane protein 1 (LAMP1)‐positive vesicles) was also markedly reduced in BNIP3 KD cells (Figure S1F). The mitophagy activation in response to incubation with carbonyl cyanide *m*‐chlorophenylhydrazone (CCCP) was also affected in these cells. Treatment of myotubes for 1 h with CCCP triggered an increase in the LC3II present in mitochondrial fractions in control cells due to the induction of mitophagy, and this effect was blocked in BNIP3 KD myotubes (Figure S1G,H). In contrast, PTEN‐induced putative kinase 1 (PINK1) abundance was enhanced both in control and BNIP3 KD cells in response to CCCP, suggesting that PINK1‐dependent mitophagy is not affected by BNIP3 deficiency in mouse muscle cells (Figure S1G,H).

To study the effect of impaired mitophagy on the downregulation of BNIP3 in mitochondrial homeostasis, we assessed various parameters of the mitochondrial function in muscle cells. The BNIP3 repression was also linked to a reduced mitochondrial membrane potential (Figure S1I) and to increased hydrogen peroxide (H_2_O_2_) production (Figure S1J). In addition, mitochondrial respiration revealed enhanced basal respiration as a result of an increased proton leak in BNIP3 KD myotubes compared to control cells (Figure 1F). The pattern of mitochondrial dysfunction was associated with mitochondrial fragmentation, as determined by live imaging confocal microscopy (Figure 1G) and transmission electron microscopy (TEM) (Figure 1H,I). In all, these data indicate that BNIP3 regulates mitophagy and sustains mitochondrial homeostasis in skeletal muscle cells.

### 2.2 Downregulation of BNIP3 impairs lysosomal function

**FIGURE 2:**
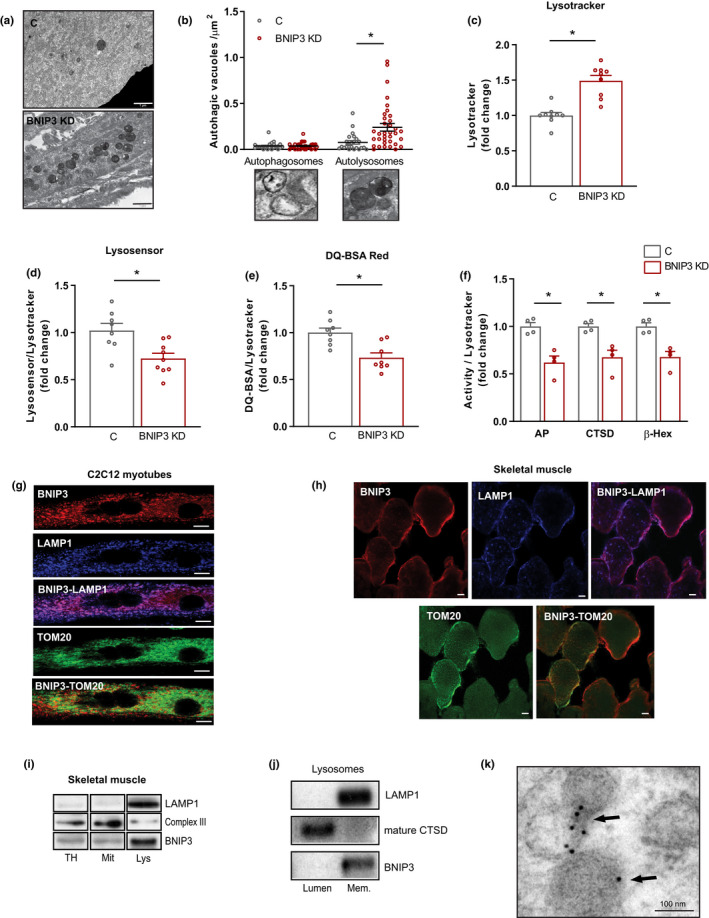
BCL2 interacting protein 3 (BNIP3) regulates lysosomal function in skeletal muscle cells. (a) Representative transmission electron microscopy (TEM) images of control (C) and BNIP3 knockdown (BNIP3 KD) cells showing accumulation of electron‐dense vesicles in BNIP3 KD myotubes (scale bar 1 µm). (b) Quantification of autophagosomes and autolysosomes per µm^2^ in TEM images from C and BNIP3 KD myotubes (*n* = 25–30 images per genotype). (c) Lysosomal mass determination by Lysotracker Red fluorescence quantification by flow cytometry in C and BNIP3 KD myotubes (*n* = 9). (d) Lysosensor Blue quantification by flow cytometry corrected by lysosomal mass in C and BNIP3 KD myotubes (*n* = 9). (e) Dye quenched‐red bovine serum albumin (DQ‐Red BSA) quantification by flow cytometry corrected by lysosomal mass in C and BNIP3 KD myotubes (*n* = 8). (f) Lysosomal enzyme activity (acid phosphatase ‐AP‐, cathepsin D ‐CTSD‐, and β‐hexominidase ‐β‐Hex‐) corrected by lysosomal mass in total homogenates from C and BNIP3 KD myotubes (*n* = 4). (g) Representative immunofluorescence images of BNIP3, lysosomal‐associated membrane protein 1 (LAMP1), and translocase of the outer membrane 20 (TOM20) in myotubes (scale bar 10 µm). (h) Representative immunofluorescence images of BNIP3, LAMP1, and TOM20 in mouse gastrocnemius muscle (scale bar 10 µm). (i) Western blot (WB) analysis of BNIP3 in different pure fractions from mouse gastrocnemius muscle. LAMP1 was used as a lysosomal maker, and complex III as a mitochondrial marker (TH, total homogenate; Mit, mitochondria; Lys, lysosomes). (J) Western blot (WB) analysis of BNIP3 in lumen and membranes of pure lysosomes. LAMP1 was used as a lysosomal membrane marker and CTSD as a lysosomal lumen marker. (k) Immunogold TEM image showing the presence of BNIP3 in lysosomes from C2C12 myotubes (scale bar 100 nm). Data are expressed as mean ± SE. *p < 0.05

Analysis of TEM images of BNIP3 KD muscle cells revealed that the repression of BNIP3 induced an accumulation of electron‐dense vesicles, corresponding to an increase in autolysosomes (Figure 2A,B). In keeping with these observations, BNIP3 KD cells showed increased expression of late endosome/lysosome markers such as Rab7 and LAMP1—but not early endosome markers such as Early Endosome Antigen 1 (EEA1) and Rab5 (Figure S2A)—, larger LAMP1‐positive vesicles (Figure S2B) and increased lysosomal mass, as shown by the quantification of Lysotracker staining (Figure 2C). Since alterations in lysosomal mass and size have been associated with lysosomal dysfunction (Fernandez‐Mosquera et al., 2019), we used a number of distinct experimental approaches to assess the lysosomal function in control and BNIP3 KD myotubes. First, the lysosomal pH was assessed by using Lysosensor Blue, a fluorescent probe that accumulates in lysosomes depending on their acidic pH. Quantification of this probe by flow cytometry showed reduced accumulation inside the lysosomes of BNIP3 KD cells, thus indicating a less acidic pH, which is commonly associated with impaired lysosomal function (Figure 2D). Second, lysosomal degradation activity was measured using dye quenched‐red bovine serum albumin (DQ‐Red BSA), which is internalized into cells through the endosomal pathway, and once it reaches the lysosome, it is processed by lysosomal hydrolases and its fluorescence is dequenched. Thus, greater fluorescence is associated with an increase in lysosomal activity. Incubation of myotubes with DQ‐Red BSA and quantification of fluorescence by flow cytometry showed that BNIP3 repression led to decreased lysosomal function (Figure 2E). Third, measurements of the activity of three different lysosomal enzymes in total homogenates of BNIP3 KD cells revealed reduced activity for all three (Figure 2F). Finally, we examined the processing of cathepsin B and D, which are synthesized as precursor proteins in the endoplasmic reticulum (ER) and processed in the lysosome by lysosomal enzymes to produce the cleaved active forms. A reduction in the active forms of cathepsin B and D was detected in BNIP3 KD cells compared to control cells (Figure S2C). In all, these data indicate that repression of BNIP3 leads to the accumulation of dysfunctional lysosomes in muscle cells. In keeping with these observations, immunofluorescence microscopy revealed a lysosomal localization of BNIP3 in myotubes and also myoblasts, as indicated by a substantial co‐distribution with the lysosomal marker LAMP1 (Figure 2G; Figure S2D). Indeed, BNIP3 distribution showed a higher co‐localization with LAMP1 than with translocase of the outer membrane 20 (TOM20) in myotubes (Figure S2E). Immunofluorescence analyses were also performed in mouse skeletal muscle sections, showing that BNIP3 also co‐localized with LAMP1 and to a lesser extent with TOM20 (Figure 2H). Moreover, BNIP3 was also present in pure lysosomal fractions obtained from mouse muscle (Figure 2I), thereby corroborating that this protein localizes in lysosomes. Interestingly, BNIP3 was located in lysosomal membranes and not in the lumen (Figure 2J). Finally, immunogold electron microscopy confirmed the presence of BNIP3 in the membrane of lysosome‐like structures in myotubes (Figure 2K). In all, our data indicate that BNIP3 is present in lysosomes, where it may play a functional role.

### 2.3 BNIP3 deficiency drives muscle TLR9‐dependent inflammation

**FIGURE 3:**
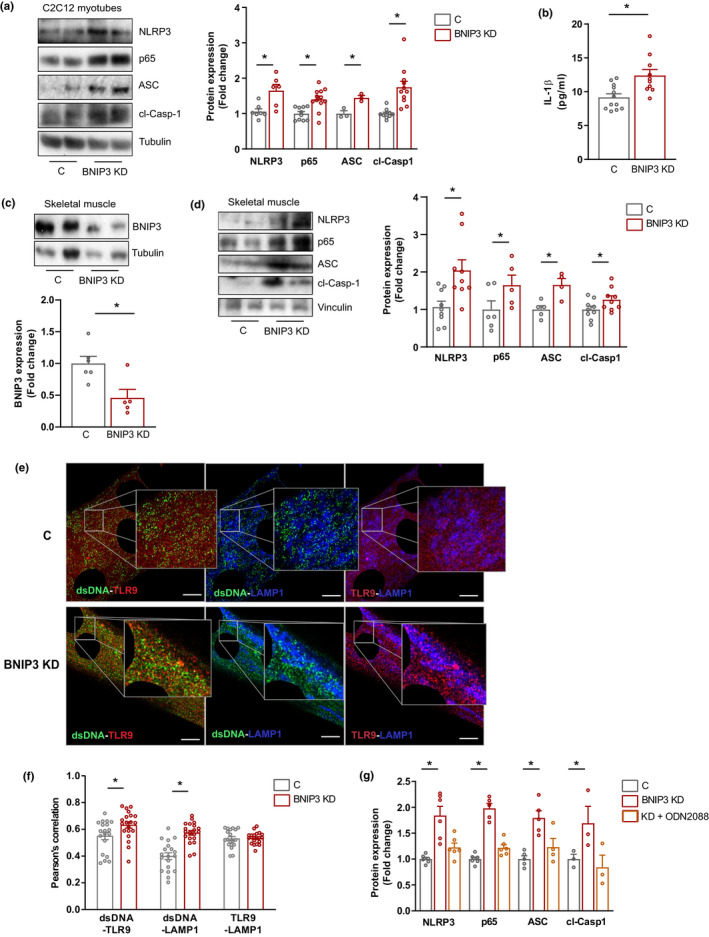
BCL2 interacting protein 3 (BNIP3) repression leads to activation of the NLRP3 (nucleotide‐binding oligomerization domain (NOD)‐, leucine‐rich repeat (LRR)‐, and pyrin domain‐containing protein 3) inflammasome and nuclear factor kappa B (NF‐κB)‐dependent inflammation in muscle. (a) Representative Western blot (WB) and quantification of NLRP3 inflammasome components and p65 protein in control (C) and BNIP3 knockdown (BNIP3 KD) myotubes (*n* = 6–9). (b) Interleukin 1β (IL‐1β) levels in culture medium from C and BNIP3 KD myotubes (*n* = 10). (c) Representative Western blot (WB) and quantification of BNIP3 protein expression in C and BNIP3 KD mouse gastrocnemius muscle (*n* = 5). (d) Representative WB and quantification of NLRP3 inflammasome components and p65 protein in C and BNIP3 KD gastrocnemius muscle (*n* = 10). (e) Representative immunofluorescence image and quantification of the co‐distribution between double‐stranded DNA (dsDNA), Toll‐like receptor 9 (TLR9), and lysosomal‐associated membrane protein 1 (LAMP1) in C and BNIP3 KD myotubes (*n* = 21 myotubes). (f) Representative WB and quantification of inflammasome components and p65 in C myotubes, and BNIP3 KD myotubes untreated or treated with TLR9 antagonist oligodeoxynucleotide (ODN) 2088 for 16 h (*n* = 6). Data are expressed as mean ± SE. *p < 0.05

Because alterations in mitophagy are associated with inflammation (Li et al., 2019; Rodriguez‐Nuevo et al., 2018; Sliter et al., 2018), we analyzed whether BNIP3 participates in muscle inflammation. BNIP3 KD myotubes showed an enhanced expression of some nuclear factor kappa B (NF‐κB) target genes but not of IRF3 target genes (responsible for type I interferon (IFN) response) (Figure S3A,B). The analysis of protein expression confirmed an upregulation of NLRP3 inflammasome components in BNIP3 KD myotubes (Figure 3A), as well as NLRP3 inflammasome activation, as indicated by increased cleaved caspase 1 (Figure 3A) and increased secretion of interleukin 1β (IL‐1β) (Figure 3B). To assess whether the pro‐inflammatory profile induced by BNIP3 repression was conserved in vivo, BNIP3 was repressed in mouse gastrocnemius muscles by adenoviral transduction of a short hairpin BCL2 interacting protein 3 (shBNIP3) construct. This approach reduced muscle BNIP3 expression (Figure 3C; Figure S3C), thus leading to increased ROS levels (Figure S3D), consistent with impaired mitochondrial homeostasis. Importantly, BNIP3 downregulation induced the same pro‐inflammatory profile as that observed in myotubes (Figure 3D), which was not associated with changes in the muscle fiber area (Figure S3E).

To decipher the processes by which BNIP3 deficiency triggers inflammation, and since BNIP3 loss‐of‐function impairs both mitochondrial and lysosomal functions, we analyzed the functional impact of either mitochondrial‐ or lysosomal‐derived damage‐associated molecular patterns (DAMPs). First, we studied the role of increased ROS levels as an inducer of myotube inflammation. Thus, we treated control and BNIP3 KD cells with the antioxidant compound N‐acetylcysteine (NAC). Although NAC reduced ROS levels in BNIP3 KD cells (Figure S4A), it was unable to significantly block the expression of inflammatory markers (Figure S4B). This observation thus suggests that increased ROS levels do not trigger muscle inflammation in BNIP3 KD conditions. Next, we examined the possible contribution of lysosomal leakage of cathepsins to inflammation induced by BNIP3 deficiency, as it has been described that such leakage to the cytosol may induce inflammation (Amaral et al., 2018). In this regard, analysis of cathepsin B in cytosolic fractions from control and BNIP3 KD cells showed the absence of this protein (Figure S4C), thereby supporting the view that cathepsin release to the cytosol does not trigger inflammation under our conditions. Finally, we turned our attention to mtDNA, a mtDAMP involved in inflammatory responses (Oka et al., 2012). Upon mitochondrial dysfunction, mtDNA can be released to the cytosol, where it activates the cGAS/STING pathway, leading to the induction of type I interferon (IFN) response (West et al., 2015). However, this pathway was not activated by BNIP3 deficiency (Figure S3B), and therefore we discarded cytosolic mtDNA as a trigger of inflammation in BNIP3 KD myotubes. Based on previous findings by our group showing that impaired mitophagy in optic atrophy type 1 (OPA1)‐deficient cells leads to TLR9‐dependent inflammation (Rodriguez‐Nuevo et al., 2018), we explored this pathway in BNIP3 KD myotubes. We performed immunofluorescence studies by using an antibody against double‐stranded DNA (dsDNA), which detected both nuclear and extra‐nuclear DNA, mainly mtDNA, as indicated by the co‐distribution of dsDNA and the mitochondrial marker TOM20 (Figure S4D). The use of this approach showed an increased co‐distribution of mtDNA and TLR9 in these cells (Figure 3E,F; Figure S4E). Importantly, this co‐localization occurred in late endosomes/lysosomes, as indicated by the increased co‐localization of mtDNA and LAMP1 in BNIP3 KD myotubes, with no changes in the amount of TLR9 co‐localizing with LAMP1 (Figure 3F), thereby indicating that enhanced mtDNA–TLR9 interaction in these myotubes is not a result of an increase of TLR9 in LAMP1‐positive vesicles. In addition, treatment with the TLR9 antagonist oligodeoxynucleotide (ODN) 2088 blocked the increased expression of NF‐κB target genes and markers of NLRP3 inflammasome activation in BNIP3 KD myotubes (Figure 3G; Figure S4F,G), suggesting that TLR9 activity is necessary for the inflammation driven by BNIP3 deficiency. Taken together, these results are coherent with a model in which BNIP3 deficiency triggers inflammation by increasing the interaction of mtDNA and TLR9 within undegraded lysosomes, thus inducing the expression of NF‐κB target genes and the activation of the NLRP3 inflammasome.

### 2.4 BNIP3 upregulation protects against muscle inflammation and atrophy during aging

**FIGURE 4:**
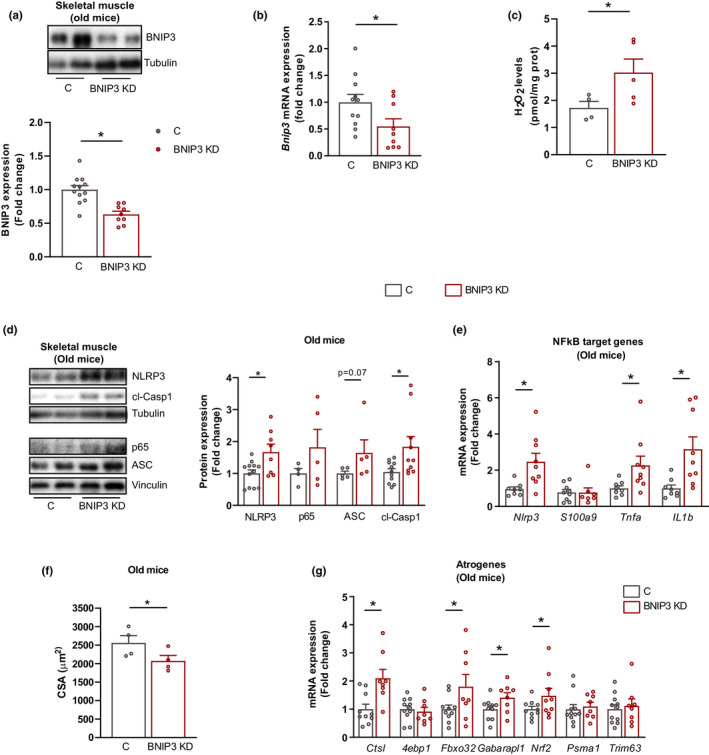
BCL2 interacting protein 3 (BNIP3) repression in old mice enhances inflammation and muscle atrophy. (a) Representative Western blot (WB) and quantification of BNIP3 protein expression in control (C) and BNIP3 knockdown (BNIP3 KD) gastrocnemius muscle from 22 to 24‐month‐old mice (old mice) (*n* = 9). (b) BNIP3 messenger RNA (BNIP3 mRNA) levels in C and BNIP3 KD gastrocnemius muscle from old mice (*n* = 8–10). (c) Hydrogen peroxide (H_2_O_2_) levels in C and BNIP3 KD muscle from old mice (*n* = 4–5). (d) Representative WB and quantification of NLRP3 (nucleotide‐binding oligomerization domain (NOD)‐, leucine‐rich repeat (LRR)‐, and pyrin domain‐containing protein 3) inflammasome components and p65 protein in C and BNIP3 KD gastrocnemius muscle from old mice (*n* = 5–8). (e) Cross‐sectional area (CSA) quantification from C and BNIP3 KD gastrocnemius muscle from old mice (*n* = 4). (f) Nuclear factor kappa B (NF‐κB) target gene messenger RNA (mRNA) expression in C and BNIP3 KD muscle from old mice (*n* = 9). (g) mRNA expression of several atrogenes in C and BNIP3 KD muscle from old mice (*n* = 9). Data are expressed as mean ± SE. *p < 0.05

Here, we document that BNIP3 deficiency triggers muscle inflammation and that muscle BNIP3 expression increases during aging. However, aging is associated with a low‐grade chronic inflammation, which is linked to aging‐related diseases and sarcopenia (Ferrucci & Fabbri, 2018). Therefore, we hypothesized that aging‐induced BNIP3 could be an adaptive mechanism to mitigate inflammation in skeletal muscle. To corroborate this notion, we analyzed the role of BNIP3 in inflammation in old mice. First, we confirmed that 24‐month‐old mice, but not 12‐month‐old counterparts, showed increased expression and activation of the NLRP3 inflammasome and increased expression of the NF‐κB subunit p65 compared with younger mice (6‐month‐old) (Figure S5A). As previously shown for young mice, adenoviral‐mediated gene transfer of shRNA reduced muscle BNIP3 expression in 24‐month‐old mice (Figure 4A,B), leading to increased ROS production (Figure 4C). Importantly, BNIP3 repression in the muscles of old animals led to an increased expression of NF‐κB target genes and inflammatory markers (Figure 4D,E). Increased inflammation during aging has been proposed to be associated with muscle atrophy and sarcopenia (Ferrucci & Fabbri, 2018). In this regard, we show that in old mice muscle BNIP3 repression reduced the muscle cross‐sectional area (CSA) (Figure 4F; Figure S5B), increased the expression of atrogenes (Figure 4G), and increased the presence of small fibers intermingled with fibers of normal size, which is characteristic of atrophic muscles (Figure S5B,C). In addition, in keeping with the fact that denervation is a well‐known contributor to muscle atrophy during aging (Larsson et al., 2019), we found that BNIP3 KD upregulated the expression of some denervation‐associated genes and increased protein abundance of the denervation protein marker neural cell adhesion molecule (NCAM) (Covault & Sanes, 1985) (Figure S5D,E). These observations thus suggest that increased inflammation associated with lower expression of BNIP3 rapidly accelerates the development of muscle atrophy in aged mice. Taken together, these data indicate that BNIP3 expression rises in skeletal muscle during aging and confers resistance to increased inflammation and muscle atrophy.

### 2.5 High muscle BNIP3 expression is associated with low inflammatory markers in aged individuals

**FIGURE 5:**
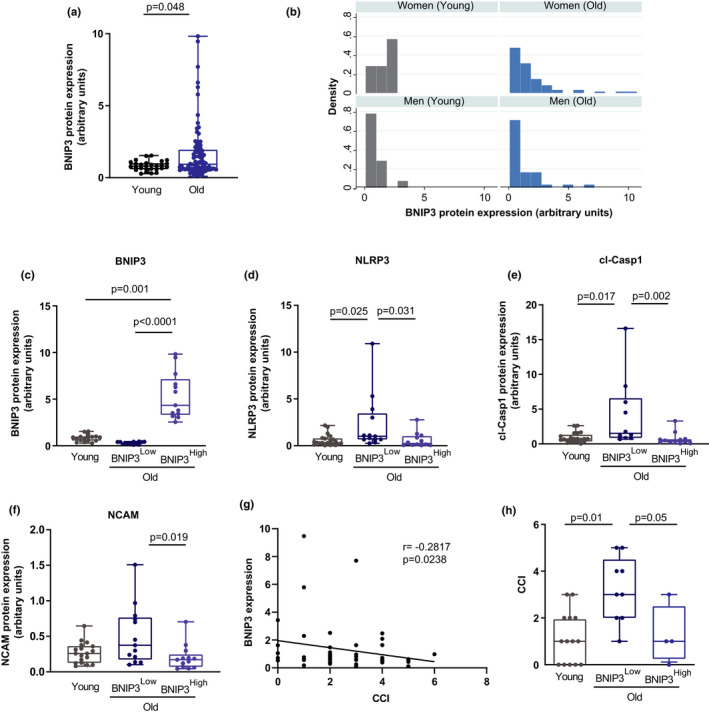
High BCL2 interacting protein 3 (BNIP3) protein expression is associated with a low inflammatory profile in aged human muscle. (a) Quantification of BNIP3 protein expression in muscle biopsies from young (*n* = 23) and old (*n* = 99) human subjects. (b) Distribution of values for BNIP3 expression in young and old subjects. (c) Quantification of BNIP3 protein levels in young subjects (*n* = 23) and old subjects with the lowest BNIP3 expression (Old BNIP3^Low^, *n* = 12) and the highest BNIP3 protein expression (Old BNIP3^High^, *n* = 12). (d–f) Expression levels of the inflammatory markers NLRP3 (nucleotide‐binding oligomerization domain (NOD)‐, leucine‐rich repeat (LRR)‐, and pyrin domain‐containing protein 3) and cleaved caspase 1, and the denervation marker neural cell adhesion molecule (NCAM), in young, Old BNIP3^Low^ and Old BNIP3^High^ subjects. (g) Correlation between Charlson comorbidity index (CCI) and muscle BNIP3 protein expression levels in old subjects (*n* = 50). (H) CCI values in young, old BNIP3^Low^, and old BNIP3^High^ subjects. Data are expressed as box and whiskers showing minimum to maximum data

Next, we analyzed whether this aging‐induced upregulation of BNIP3 was also present in human muscle biopsies obtained from young and aged subjects. Muscle biopsies (gluteal muscle) were obtained from subjects undergoing surgery involving either hip arthroplasty or hip fracture. The anthropometric parameters of the participants are summarized in Table S1. Average BNIP3 protein expression was increased in aged subjects (Figure 5A). In addition, the distribution of values was different between the young and aged group, with aged group ranging from low to very high levels of BNIP3 expression, with no differences between women and men (Figure 5A,B). Therefore, the relationship between age and BNIP3 expression was analyzed by bivariate and multivariate regression models (Table S2). Although there was a positive correlation between BNIP3 expression and age in the bivariate analysis, no statistically significant association between age and BNIP3 was detected either in the bivariate or in the multivariate regression model, when adjusted by gender or body mass index (BMI) (Table S2). Since aged subjects showed a high variability in BNIP3 expression, and to gain insight into the physiological role of BNIP3 and inflammation in aging muscles, we measured the inflammatory markers in human muscle biopsies showing the extreme BNIP3 levels. Hence, we categorized aged subjects in the group with the lowest BNIP3 expression (Old BNIP3^Low^, with BNIP3 levels <50% of the mean value for BNIP3 expression in old subjects), and with the highest BNIP3 expression (Old BNIP3^High^, with BNIP3 levels >2‐fold of the mean value for BNIP3 expression in old subjects) (Figure 5C), and we analyzed NLRP3 and cleaved caspase 1 protein expression as markers of inflammation. Aged subjects with low BNIP3 expression showed increased NLRP3 and cleaved caspase 1 compared to young subjects and to aged subjects with high BNIP3 expression (Figure 5D,E). Of note, aged subjects with high BNIP3 expression showed levels of NLRP3 and cleaved caspase 1 indistinguishable from young subjects. In addition, the denervation marker NCAM was decreased in aged subjects with high BNIP3 expression (Figure 5F). In order to assess whether BNIP3 expression levels were associated with healthy aging and probability of developing sarcopenia, we used the Charlson comorbidity index (CCI), a method that categorizes comorbidities of patients based on the International Classification of Diseases, which has been associated with skeletal muscle mass and physical performance in aged individuals (Gong et al., 2019). CCI correlated negatively with BNIP3 expression in aged subjects (Figure 5G), and interestingly, the group of aged subjects with high BNIP3 levels showed a significant decrease in this parameter (Figure 5H). These results suggest that, as documented in mice, increased BNIP3 protein expression during aging protects against muscle inflammation and denervation, which could be associated with a higher muscle mass, physical performance, and a healthy aging in humans.

## 3 DISCUSSION

Here, we document a key role of BNIP3 in the maintenance of mitochondrial quality control, lysosomal function, inflammation, and homeostasis in skeletal muscle. We also demonstrate that muscle BNIP3 expression increases in humans and mice during aging and that this increase protects against aging‐induced inflammation. This conclusion is based on a number of relevant observations, namely: (i) normal aging in mouse is characterized by increased BNIP3 expression in skeletal muscle; (ii) repression of muscle BNIP3 in aged mice induces inflammation; (iii) high BNIP3 expression in aged human skeletal muscle is associated with a low inflammatory profile; (iv) repression of BNIP3 in myotubes leads to inhibition of mitophagy, lysosomal dysfunction, and mitochondrial damage; and (v) BNIP3 repression leads to TLR9‐dependent activation of NF‐κB, which triggers inflammation.

In mouse, decreased mitophagy in various tissues is associated with age‐related diseases (Bravo‐San Pedro et al., 2017; Garcia‐Prat et al., 2016; Sebastian et al., 2016), and interventions designed to increase mitophagy have been proposed to counteract some of the alterations linked to aging in model organisms and humans (Andreux et al., 2019; Fang et al., 2019; Ryu et al., 2016). In our previous work, we showed that during aging there is a reduction of Mfn2 protein in mouse muscle, which leads to inhibition of mitophagy and is associated with age‐induced metabolic disease and sarcopenia (Sebastian et al., 2016). Alterations in some markers of mitophagy have also been found to be associated with aging and age‐related diseases in humans (Babbar et al., 2020; Castellazzi et al., 2019), and with longevity in several model organisms, including *Caenorhabditis elegan*s (*C*. *elegans*) and *Drosophila melanogaste*r (*D*. *melanogaster*) (Aparicio et al., 2019; Palikaras et al., 2015; Schiavi et al., 2015). In particular, Palikaras et al. (2015) reported that, in *C*. *elegans*, mutants lacking dct‐1, the ortholog of NIX, another member of the BH_3_‐only domain proteins of the BCL_2_ family like BNIP3, showed accelerating aging, thus pointing to a key role of this protein in aging.

Our data show that BNIP3 downregulation in mouse muscle leads to an increase in age‐related phenotypes such as muscle atrophy and inflammation. Interestingly, although activation of NF‐κB and inflammation induce muscle atrophy in young mice (Cai et al., 2004), here we report that inflammation associated with BNIP3 repression is linked to muscle atrophy in old mice but not in young counterparts. This discrepancy could be explained, either by differences in the study model (chronic vs. acute upregulation of NF‐κB activity) or by a specific role of the inflammation caused by BNIP3 deficiency in triggering muscle atrophy only in the context of aging. In keeping with this, we show that downregulation of BNIP3 in old mice leads to an increase in denervation, which is a key contributor to muscle atrophy with aging (Larsson et al., 2019). Since we have used the injection of adenoviral vectors to repress BNIP3, we cannot conclude whether the decrease in BNIP3 is affecting the presynaptic or postsynaptic side of the endplate. However, given the effect of BNIP3 downregulation inducing inflammation in our cell culture experiments, we propose that a cell autonomous effect of this protein within the muscle fiber is key to promoting inflammation and probably the degeneration of the neuromuscular junction, leading to muscle atrophy. Future work using skeletal muscle specific ablation of BNIP3 would be necessary to address this question in detail.

In humans, we show that high levels of BNIP3 are associated with lower muscle inflammation and denervation markers in aged subjects. However, whether this correlates with muscle atrophy has not been directly addressed, since muscle mass/fiber area measurements were not available for our human cohort, which is a limitation of our study. However, the analysis of the denervation marker NCAM shows that high BNIP3 expression protects from denervation, which could be associated with a lower muscle atrophy during aging. Interestingly, increased muscle BNIP3 levels are associated with a lower number of comorbidities, as indicated by a lower Charlson comorbidity index (CCI). This parameter has been shown to correlate with muscle mass and physical performance in aged subjects, being a good predictor for developing sarcopenia (Gong et al., 2019). Thus, our data using the CCI suggest that high BNIP3 expression could be linked to a higher muscle mass, physical performance, and healthy aging in humans. All these results allow us to propose that the upregulation of BNIP3 during aging could be a stress response following the accumulation of unhealthy mitochondria, which would limit muscle inflammation, denervation, and atrophy. Similarly, a BNIP3‐induced stress response has also been shown to be activated in response to the metabolic stress following hypoxia and increased mitochondrial damage after exercise (Bellot et al., 2009; Zhang et al., 2008).

To study the role of BNIP3 in the maintenance of muscle homeostasis, we repressed its expression in C2C12 myotubes. As expected, BNIP3 downregulation led to the inhibition of mitophagy and thus the accumulation of dysfunctional mitochondria. However, unexpectedly, BNIP3 KD myotubes showed an increase in autolysosomes, which was not a consequence of either the increased formation of autophagosomes, as indicated by no alteration in their number, or in early steps of the endosomal pathway, as indicated by no changes in early endosomal markers. However, we observed an increase in late endosomal/lysosomal markers and enlarged LAMP1‐positive vesicles, which, as previously shown (Fernandez‐Mosquera et al., 2019), are associated with lysosomal dysfunction. All these data strongly suggest that BNIP3‐induced lysosomal dysfunction caused the accumulation of the autolysosomes because they were unable to degrade their cargo and then be recycled.

As a possible explanation for the role of BNIP3 in lysosomal function, we found that BNIP3 was present in lysosomes, as indicated by a number of different approaches in muscle cells and skeletal muscle in vivo. Part of BNIP3 co‐localized with LAMP1 in skeletal muscle cells and gastrocnemius muscle from mice and was present in the membrane of purified lysosomes from skeletal muscle. In addition, immunogold electron microscopy in myotubes revealed the presence of BNIP3 in lysosomes. In this regard, BAX, another member of the BCL_2_ gene family, immunolocalizes in lysosomes in a variety of cell lines exposed to pro‐apoptotic stimuli (Feldstein et al., 2006; Oberle et al., 2010; Werneburg et al., 2007) and in experimental models of Parkinson's disease (Bove et al., 2014), where it has been shown to induce lysosomal membrane permeabilization and lysosomal dysfunction. In addition, mutations in ATP13A2, a lysosomal type 5 P‐type ATPase involved in Parkinson's disease, lead to lysosomal alterations very similar to those observed in BNIP3 KD myotubes, which include impaired lysosomal acidification, decreased lysosomal proteolytic activity, and accumulation of autophagolysosomes (Dehay et al., 2012). Interestingly, deletion of ATP13A2 triggers inflammation by cytosolic leakage of mtDNA in cellular and zebrafish models of Parkinson's disease, thus suggesting a link between lysosomal dysfunction and inflammation (Matsui et al., 2021). Although further experiments would be necessary to decipher the specific role of BNIP3 in lysosomes, our findings support the notion that BNIP3 participates in the regulation of lysosomal function beyond its role as a mitophagy receptor, which would be important for triggering inflammation.

Alterations in mitophagy are associated with inflammation in various models (Li et al., 2019; Rodriguez‐Nuevo et al., 2018; Sliter et al., 2018). Since mitochondria can be a source of DAMPs, mitochondrial abnormalities caused by defects in mitophagy could trigger inflammatory responses (Riley & Tait, 2020; Rodriguez‐Nuevo & Zorzano, 2019). Here, we show that the repression of BNIP3 in muscle cells and mouse muscle activates the NLRP3 inflammasome and induces NF‐κB‐dependent inflammation, and detailed mechanistic studies demonstrate that an increase in mtDNA–TLR9 interaction in LAMP‐1 positive vesicles causes this increased inflammation. Under basal conditions, TLR9 resides in the ER, but recognition of DNA occurs in the endosomes or lysosomes, leading to the recruitment of MyD88, which activates NF‐κB, inducing an inflammatory response (De Leo et al., 2016; Latz et al., 2004). Therefore, alterations in mitophagy, autophagy, or autophagosome–lysosome fusion, which can lead to an increase in nondegraded mtDNA, have been associated with TLR9 activation (De Leo et al., 2016; Oka et al., 2012; Rodriguez‐Nuevo et al., 2018). In keeping with this, our results suggest that the alterations in mitophagy and lysosomal function caused by the decrease in BNIP3 lead to the accumulation of autolysosomes with undegraded cargo, which favors the interaction of mtDNA and TLR9 leading to the induction of NF‐κB and inflammation.

As discussed above, alterations in mitophagy, accumulation of dysfunctional mitochondria, and inflammation are proposed to participate in aging and age‐related diseases (Babbar et al., 2020; Ferrucci & Fabbri, 2018), and mitophagy defects and accumulation of damaged mitochondria induce inflammation (Li et al., 2019; Rodriguez‐Nuevo et al., 2018; Sliter et al., 2018). However, a direct connection between alterations in mitophagy, the accumulation of dysfunctional mitochondria, and inflammaging has not been reported to date. In this regard, we propose that BNIP3 is a key protein in the maintenance of mitochondrial health by regulating mitophagy and lysosomal function, and that its adaptive increase during aging in a context of a general decrease in mitophagy mitigates mitochondrial damage and confers resistance to age‐induced inflammation and muscle atrophy, thus establishing a direct link between loss of mitochondrial homeostasis and age‐induced inflammation. On the basis of our results, interventions enhancing BNIP3‐regulated mitochondrial health emerge as a promising strategy to prevent muscle inflammaging and sarcopenia.

## 4 EXPERIMENTAL PROCEDURES

### 4.1 Human muscle biopsies

Muscle biopsies (gluteal muscle) were obtained under spinal anesthesia and sedation from patients operated either for coxarthrosis (total hip arthroplasty) or for hip fracture (synthesis using a trochanteric endomedullary nail or dynamic hip screw—in the case of extraarticular fractures— or hemiarthroplasty—in the case of intraarticular fractures‐) at the Fundació Sanitaria Parc Sant Joan de Déu. Subjects suffering from either myopathy or peripheric polyneuropathy, or treated with corticoids were excluded. Charlson comorbidity index (CCI) was used for comorbidity assessment and calculated as described (Charlson et al., 1987). Upon collection, 100 mg of muscle biopsy was cleaned of any visible blood and fat and then snap‐frozen in liquid nitrogen and stored at −80°C until further processing and analysis. A total of 124 biopsies were collected. All patients enrolled in this study gave their written consent to participate. All the protocols were approved by the Ethical Committee of the Fundació Sanitaria Parc Sant Joan de Déu.

### 4.2 Animals

Male C57BL6/J mice aged 4–6 months and 22–24 months were used in this study. Mice were kept in 12‐hour dark–light periods in a specific pathogen‐free (SPF) animal facility and provided with a standard chow diet and water ad libitum. At the indicated time, mice were anesthetized using isoflurane and sacrificed by cervical dislocation. All animal experiments were done in compliance with the guidelines established by the Institutional Animal Care and Use Committee of the Barcelona Science Park and University of Barcelona.

### 4.3 Cell culture, treatments, and adenoviral transduction in cells and mouse muscle

C2C12 myoblasts were used in this study. C2C12 myoblasts were differentiated as described (Sebastian et al., 2016). C2C12 myotubes were transduced with control and mouse BNIP3–shRNA adenoviruses (Ad‐GFP‐U6‐mBNIP3‐shRNA, Vector Biolabs) for 36 h at day 4 of differentiation (100 pfu (plaque‐forming units)/cell). All the experiments were performed 48 h after infection. C2C12 myotubes were incubated with bafilomycin A (200 nM), N‐acetylcysteine (5 mM), CCCP (1 μM), or ODN 2088 (1 μM), as indicated in the figure legends.

Adenoviral transduction in young and old mice was performed by injecting purified control and shBNIP3 adenoviruses at 2 × 10^11^ IU (infection units)/ml in a total volume of 75 μl into gastrocnemius muscle. Three different areas of gastrocnemius muscle were injected to assure the maximal transduction efficiency. Control adenoviruses were injected in the right muscle and shBNIP3 adenoviruses in the left muscle. Muscles were collected 1 week after adenoviral administration.

### 4.4 Protein extraction and Western blotting

Proteins from total homogenates and skeletal muscle fractions were dissolved in 10%, 12.5%, or 15% acrylamide gels for sodium dodecyl sulfate–polyacrylamide gel electrophoresis (SDS–PAGE) and transferred to Immobilon membranes (Millipore). Specific details of protein extraction from cells and muscle fractions and the list of antibodies used can be found in Data S1.

### 4.5 Determination of lysosomal enzyme activity

Lysosomal enzymatic activity was measured in 10 μg of cell homogenate from C2C12 myotubes. Further details can be found in Data S1.

### 4.6 Interleukin 1β (IL‐1β) measurement

Interleukin 1β (IL‐1β) concentration in culture media was measured using the Il1b (mouse) ELISA kit (Abnova) and following the manufacturer's instructions. Prior to the assay, culture mediums from C2C12 myotubes were concentrated using Amicon Ultra‐4 centrifugal filter units (UFC801024), following the manufacturer's instructions.

### 4.7 Immunofluorescence

Cells and optimal cutting temperature (OCT) compound‐embedded gastrocnemius were fixed in 4% paraformaldehyde (PFA) in phosphate‐buffered saline (PBS) for 20 min and washed for 10 min with 50 mM ammonium chloride (NH4Cl) and 20 mM glycine PBS. Specific details of permeabilization, antibody incubation, image acquisition, processing, and quantification can be found in Data S1.

### 4.8 Mitochondrial morphology assessment by live cell confocal microscopy

Cells were seeded in a 22‐cm diameter coverslip, differentiated, and transduced, as described above. On the day of the assay, cells were incubated with 50 nM Mitotracker Deep Red for 30 min and washed twice with PBS 1X. Fresh medium was added to the cells and coverslips were visualized using a spectral confocal microscope (Zeiss LSM 780+FLIM).

### 4.9 Histological sample preparation and analysis

For light microscopy, muscles were removed and fixed in formalin. Ten micrometer cryosections of gastrocnemius muscles were used. Sections were stained with hematoxylin and eosin (H&E) following standard protocols to check tissue architecture and cross‐sectional area (CSA). CSA was quantified in 200–250 fibers/mouse using Image J software.

### 4.10 Determination of mitochondrial oxygen consumption

The respiration of cultured muscle cells was determined using the Agilent Seahorse XF‐24 Extracellular Flux Analyzer (Seahorse Bioscience, Agilent Technologies), as previously described (Sebastian et al., 2016).

### 4.11 Measurement of reactive oxygen species (ROS)

The H_2_O_2_ levels were measured in muscle and cell homogenates using Amplex Red (Thermo Fisher), as described previously (Sebastian et al., 2016).

### 4.12 Determination of mitochondrial and lysosomal parameters by flow cytometry

Cells were incubated with the indicated probe for 30 min, washed twice with PBS 1X, and trypsinized. Fluorescence was quantified by flow cytometry in a Gallios flow cytometer (Beckman Coulter) or a BD FACS Aria SORP (Becton Dickinson). For the determination of mitochondrial membrane potential, 50 nM tetramethylrhodamine methyl ester (TMRM) (Thermo Fisher) was used. For the measurement of lysosomal mass and functionality, 50 nM Lysotracker Deep Red, 1 μM Lysosensor Blue, or 1 μg/ml DQ‐Red BSA (Thermo Fisher) was used.

### 4.13 Determination of mitochondrial mass and mitophagic flux by Mitotracker Deep Red

C2C12 myotubes were incubated for 30 min with 50 nM Mitotracker Deep Red (Thermo Fisher), washed twice with PBS 1X, and trypsinized. Mitotracker Deep Red fluorescence was quantified by flow cytometry in a Gallios cytometer (Beckman Coulter). Mitophagic flux was analyzed by the quantification of mitochondrial mass with or without bafilomycin A treatment. Myotubes were incubated for 30 min with Mitotracker Deep Red and washed with PBS 1X. They were then incubated with or without bafilomycin A (200 nM) for 16 h and the mitochondrial mass was assessed by flow cytometry, as described above.

### 4.14 Mitochondrial DNA copy number

Genomic DNA was isolated from cultured cells by using the Genelute Mammalian Genomic DNA Kit (Sigma), following the manufacturer's instructions. DNA was amplified with specific oligodeoxynucleotides for mitochondrial DNA or nuclear DNA (see Data S1). We calculated the mitochondrial DNA copy number per cell using glyceraldehyde 3‐phosphate dehydrogenase (GAPDH) amplification as a reference for nuclear DNA content.

### 4.15 Transmission electron microscopy and immunolabeling

Samples were prepared and processed in the Electron Cryomicroscopy Unit at the CCiTUB, University of Barcelona. Further details can be found in Data S1.

### 4.16 DNA and RNA extraction and real‐time PCR

RNA from cells was extracted by using PureLink RNA Mini Kit (Invitrogen) following the manufacturer's instructions. Mice were sacrificed by cervical dislocation and tissues were immediately frozen. RNA was isolated by using the Trizol reagent followed by purification with PureLink RNA Mini Kit (Invitrogen). Further details and the list of primers used can be found in Data S1.

### 4.17 Statistical analysis

The data presented here were analyzed using an appropriate normality test to assess whether the data fit a Gaussian distribution. An *F* test of equality of variances was performed to demonstrate that the variance between groups was not different. Statistical significance was determined using the Student *t* test or analysis of variance (ANOVA) with an appropriate post hoc test. Data are presented as mean ± SEM. Significance was established at *p *< 0.05.

In the study with human samples, some patients presented missing values in the independent variables (age = 3; BMI = 14) and were excluded from the analysis. In order to evaluate the potential impact of age on the expression of BNIP3, a bivariate linear regression model was fitted, where BNIP3 was the dependent variable and age was the independent variable. To control for the effect of gender and BMI, which were not equally distributed across age groups, a multivariate linear regression model was fitted where BNIP3 was the dependent variable and age, gender, and BMI were the independent variables. The *p* value and 95% confidence interval (CI) were estimated. Finally, the distribution of BNIP3 across age group and gender was represented in a histogram. For correlation analyses, a Spearman test was performed.

## CONFLICT OF INTEREST

The authors declare no conflict of interest.

## AUTHOR CONTRIBUTIONS

AI conceived and performed experiments and revised the manuscript; MMV performed experiments and revised the manuscript; PA, CA, EA, RGV, and J.C. recruited the patients participating in the study and obtained muscle biopsies; MRV performed statistical analysis for human studies; LL coordinated the clinical studies; AG discussed results and revised the manuscript; MP contributed with reagents, discussed results, and revised the manuscript; AZ conceived experiments, directed the research, revised the experimental data, and wrote the manuscript; DS conceived and performed experiments, revised the experimental data, directed the research, and wrote the manuscript.

## Supporting information

Data S1

Fig S1‐S5

## Data Availability

The data that support the findings of this study are available from the corresponding author upon reasonable request.
