# Supplementary material for: Coordination of mitochondrial and lysosomal homeostasis mitigates inflammation and muscle atrophy during aging
Source: Aging Cell. 2022 Mar 9;21(4):e13583. doi: 10.1111/acel.13583 (PMC9009131; doi:10.1111/acel.13583)
Supplement: Supplementary file 1 — Data S1 [file ACEL-21-e13583-s001.pdf]

SUPPORTING INFORMATION

Experimental procedures

Protein extraction and western blotting

Cell culture homogenates for Western Blot analyses were obtained by collecting the cells in ice-cold PBS 1X. Cells were homogenized with a 25G-syringe in lysis buffer [50 mM Tris (pH 7.5), 150 mM NaCl, 1 mM EDTA, 2 mM sodium orthovanadate, 100 mM NaF, 20 mM sodium pyrophosphate, 1% NP-40, and protease inhibitors Mini protease tablet (Roche)] and centrifuged at 700 x *g* for 10 min at 4ºC to remove nuclei, cell debris and floating cells. Mouse gastrocnemius and human muscle biopsies were homogenized in lysis buffer with a mini-beadbeater (Biospec) twice for 30 sec, incubated for 1 h at 4ºC in an orbital shaker, and then centrifuged for 15 min at 10,000 x *g*. Supernatants were aliquoted and kept at -20ºC.

Mitochondria-enriched fractions from cell cultures were obtained by homogenization of a 10-cm plate of cells using a Douncer homogenizer with a Teflon pestle in homogenization buffer (0.25M sucrose, 50 mM KCl, 5 mM EDTA, 1 mM sodium pyrophosphate, 5 mM MgCl2, pH 7.4 and protease inhibitors tablet, Roche). Homogenates were centrifuged at 740 x *g* for 5 min at 4ºC. Supernatant was centrifuged at 9,000 x *g* for 15 min at 4ºC. The supernatant was the cytosolic fraction, and the pellet (mitochondria-enriched fraction) was washed in homogenization buffer and resuspended in lysis buffer.

Purified lysosomal fractions from mouse skeletal muscle were obtained from a pool of 8 gastrocnemius muscles. Briefly, muscles were homogenized in 250 mM sucrose and the light mitochondrial-lysosomal enriched fraction was obtained after differential centrifugation. Pure lysosomal and mitochondrial fractions were isolated from this enriched fraction after discontinuous Nycodenz (Progen Biotechnik) density gradient centrifugation, as previously described (Cuervo, Dice, and Knecht 1997) . Lysosomal luminal contents and membranes were isolated from pure lysosomal fractions after five cycles of hypotonic shock and freeze/thaw cycles and centrifugation at 100,000 x *g* for 30 min.

The following antibodies were used: α-Tubulin (1/8000, Sigma); β-Actin (1/5000, Sigma); Vinculin (1/5000, Abcam); GAPDH (1/5000, ThermoFisher Scientific); BNIP3 (1/1000, Abcam); TIM44 (1/2000, BD Transduction Laboratories); NLRP3 (1/1000, Adipogen); ASC (1/500, SantaCruz Biotechnology); p65 (1/500, SantaCruz Biotechnology); caspase 1 (1/1000, Novus Biologicals); LAMP1 (1/1000, SantaCruz Biotechnology); Cathepsin B (1/1000, Calbiochem); Cathepsin D (1/1000, SantaCruz Biotechnology); Complex III subunit core 1 (Uqcrc1, 1/5000, ThermoFisher Scientific);

Complex II (Sdha, 1/5000, ThermoFisher Scientific); LC3 (1/1000, MBL); PINK1 (1/1000, Novus Biologicals); Rab7 (1/1000, Cell Signaling); Rab5 (1/1000, Cell Signaling); and EEA1 (1/1000, Cell Signaling); NCAM H-28 (1/1000). Anti-mouse, anti-rabbit, and anti-rat HRP conjugated (Jackson Laboratories) were used as secondary antibodies.

Immunofluorescence

Cells were permeabilized in 0.1% Triton X-100, 3% FBS in PBS for 30 min and then coverslips were incubated for 10 min in buffer A (0.05% Saponin, 2% FBS in PBS). Coverslips were incubated in primary antibody diluted in buffer A (1:400) for 60 min, washed with buffer A, incubated in secondary antibody diluted in FBS 10% in PBS1X (1:200) for 60 min and washed with buffer A. Cells were then washed with PBS before mounting the coverslips on microscope slides with Flouromount (Sigma).

Gastrocnemius muscles were removed, embedded in OCT solution (TissueTek), immediately frozen in liquid nitrogen-cooled isopenthane (Sigma), and stored at -80°C. 10 µm cryosections of this tissue were fixed in 4% paraformaldehyde (PFA) in PBS for 20 min and washed twice with PBS 1X for 5 min. They were then incubated with 50 mM NH4Cl and 20 mM glycine PBS 1X for 10 min each. After washing three times in PBS 1X, sections were permeabilized in 0.1% Triton X-100, 0.1 % sodium citrate in PBS 1X for 30 min and washed twice with PBS 1X for 5 min. After blocking in PBS 1X 10% FBS for 30 min, sections were incubated in primary antibody diluted in blocking solution (1:200) for 60 min, and with secondary antibody (1:200) for 60 min. Coverslips were washed three times with PBS 1X and mounted on microscope slides with Fluoromount (Sigma).

Confocal images were obtained using a spectral confocal microscope Zeiss LSM 780 with a PlanAchromat 63x oil objective lens with 1.40 NA, a pinhole diameter of 1 airy unit and a pixel size as small as 100 nm to meet Nyquist sampling criterion (to preserve the spatial resolution in the given image). Z-stacks were acquired with the optimal step. For co-localization experiments between dsDNA-TLR9-LAMP1, confocal microscope images were obtained using the Airyscan detector of a ZEISS Elyra 7 super-resolution microscope at x64 magnification. The primary antibodies used included Complex IV subunit I (COX1, ThermoFisher); LC3 (MBL), BNIP3 (Abcam), LAMP1 (SantaCruz Biotechnology), TOM20 (SantaCruz Biotechnology), dsDNA (Abcam), and TLR9 (SantaCruz Biotechnology). Alexa Fluor Pacific Blue, Alexa Fluor 568 and Alexa Fluor 647 were used as secondary antibodies.

Image processing and quantification were performed using ImageJ software. LC3-COX1 and LAMP1-TOM20 co-localization was calculated as the percentage of positive fluorescence of each

individual maker co-localizing with the other marker. mtDNA-TLR9-LAMP1 co-localization was performed with JACoP plugin (Bolte and Cordelieres 2006), establishing the same thresholds for each plugin in each image. All quantifications were done on stacks of images.

Mitochondrial DNA copy number

The following primers were used to amplify mtDNA: specific primers to amplify mouse mitochondrial DNA in positions 1,212 and 1,352: Fwd ACCGCAAGGGAAAGATGAAAG, and Rev AGGTAGCTCGTTTGGTTTCGG; and the primers used to amplify nuclear DNA (GAPDH) were: Fwd: CATGGCCTTCCGTGTTCCTA, Rev: GCGGCACGTCAGATCCA.

Determination of lysosomal enzyme activity

β-Hexosaminidase activity was measured using 4 mM 4-methyllumbelliferyl-N-actetyl-B-D-glucopyranoside (Sigma) as substrate in acetate buffer at pH 4.4 with 0.25% Triton X-100 in a 96-well plate incubated at 37°C for 1 h. The reaction was stopped with stop solution (2 M glycine, 2 M Na2CO3) and fluorescence was measured at ex360/em460 nm. Acid phosphatase (AP) activity was measured using 4-Nitrophenyl phosphate disodium (Sigma) as substrate in citrate buffer at pH 4.5 in a 96-well plate incubated at 37°C for 1 h. The reaction was arrested with stop solution (0.5N NaOH) and absorbance was measured at 405 nm. Cathepsin D (CTSD) activity was determined using the Cathepsin D Activity Assay (Abcam), following the manufacturer's instructions.

Transmission electron microscopy and immunolabeling

C2C12 myotubes were washed twice with 0.1 M phosphate buffer at room temperature. For fixation, cells were incubated in 2.5% glutaraldehyde solution at room temperature for 70 min. Following post-fixation in 1% osmium tetroxide in 0.1 M phosphate buffer at 4°C for 2 h, cells were washed with highly pure water and kept overnight in 0.1 M phosphate buffer. Next, the samples were dehydrated in acetone, infiltrated with Epon resin for 2 days, embedded in the same resin orientated for longitudinal sectioning and polymerized at 60°C for 48 h. When found, ultrathin sections were obtained using a Leica Ultracut UC6 ultramicrotome (Leica Microsystems, Vienna, Austria) and mounted on Formvar-coated copper grids. They were stained with 2% uranyl acetate in water and lead citrate. Sections were then observed under a JEM-1010 electron microscope (Jeol, Japan) equipped with a CCD camera SIS Megaview III and the AnalySIS software. Mitochondrial morphology was assessed by quantifying the aspect ratio of 350-400 mitochondria per condition. Number of autophagosomes and autolysosomes were quantified in 25-30 different fields.

For immunolabeling experiments, samples were cryoimmobilized using a Leica HPM100 High-Pressure Freezer (Leica Microsystems, Vienna, Austria). Planchettes containing the frozen samples were transferred to cryotubes containing 0.5% uranyl acetate (EMS, Hatfield, USA) in acetone under liquid nitrogen and were freeze substituted at -90°C for 80 h in an EM AFS2 (Leica Microsystems, Vienna, Austria). Samples were warmed up to -50°C at 5°C/h slope and kept at -50°C. They were rinsed with acetone and infiltrated in Lowicryl HM20 resin (EMS, Hatfield, USA) at -50°C. Samples were polymerized under UV light: at –50°C for 24 h during the warming up at 5 ^{o}C/h slope until 22°C and at 22°C for 48 h. Sections of 60 nm in thickness were obtained using a UC6 ultramicrotome (LeicaMicrosystems, Vienna, Austria). They were washed sequentially in 10 mM PBS, glycine 10 mM and 10 mM PBS and then incubated on drops of 5% bovine serum albumin (BSA) in 10 mM PBS for 15 min. They were changed to 1% BSA in 10 mM drops, followed by incubation with BNIP3 antibody (Abcam) 1:5 in 10 mM PBS for 1 h. Next, they were washed in 0.25% Tween 20 in 10 mM PBS and changed to 1% BSA in 10 mM PBS, followed by incubation in 12 nm anti-mouse 1:30 (Jackson) in 1% BSA 10 mM PBS for 30 min. Samples were washed in PBS, incubated in 1% glutaraldehyde in PBS for 5 min and rinsed in MilliQ water. As a negative control for non-specific binding of the colloidal gold-conjugated antibody, the primary polyclonal antibody was omitted. Sections were stained with 2% uranyl acetate and lead citrate and were observed in a Jeol 1010 (Gatan, Japan), equipped with a tungsten cathode, in the Electron Cryomicroscopy Unit at the CCiTUB. Images were acquired at 80 kv with a CCD Megaview 1kx1k.

DNA and RNA extraction and real time-PCR

RNA was reverse-transcribed by using the Q-Script cDNA SuperMix (QuantaBio). Quantitative real-time PCR was performed using the QuantStudio 6 Real-Time PCR system (ThermoFisher) and the SYBR® Green PCR Master Mix (Applied Biosystems). All measurements were normalized to *β-actin* and *Gapdh*. The following Sybr Green primers were used:

β-actin, Fwd: GGTCATCACTATTGGCAACGA, Rev: GTCAGCAATGCCTGG
Gapdh: Fwd: CATGGCCTTCCGTGTTCCTA, Rev: GCGGCACGTCAGATCCA
Bnip3, Fwd: CACCTTTATCACTCTGCTGAATTCTCT, Rev: GATTTTGTTTTTCATTTCCAGTCTTTTAA
Nlrp3, Fwd: CGAGACCTCTGGGAAAAAGCT, Rev: CATACCATAGAGGAATGTGATGTACA
Asc, Fwd: GAAGCTGCTGACAGTGCAAC, Rev: GCCACAGCTCCAGA CTCTTC
S100a9, Fwd: TGAGCAAGAAGGAATTCAGACAAA, Rev: TGTGTCCAGGTCCTCCATGA
Rage, Fwd: GAAGGCTCTGTGGGTGAGTC, Rev: CCGCTTCCTCTGACTGATTC
Hmgb1, Fwd: CGCGGAGGAAAATCAACTAA, Rev: TCATAACGAGCCTTGTCAGC
Myd88, Fwd: GAAACTCCACAGGCGAGCGTA, Rev: GTTAAGCGCGAC CAAGGGTATG
Tnfa, Fwd: CACAAGATGCTGGGACAGTGA, Rev: TCCTTGATGGTGGTGCATGA
Il-6, Fwd: GCCCACCAAGAACGATAGTCA, Rev: CAAGAAGGCAACTGGATGGAA

Il-1b; Fwd: GCAACTGTTCCTGAACTCAACT, Rev: ATCTTTTGGGGTCCGTCAACT
Ifnb, Fwd: CCCTATGGAGATGACGGAGA, Rev: CCCAGTGCTGGAGAAATTGT
Usp18, Fwd: AGAGTTAGCAAGCTCCGACAT, Rev: TGAGGTGAATGGTCAAGGTTTG
Ifit1, Fwd: CTGAGATGTCACTTCACATGGAA, Rev: GTGCATCCCCAATGGGTTCT
Irf7, Fwd: CAATTCAGGGGATCCAGTTG; Rev: AGCATTGCTGAGGCTCACTT
Stat1, Fwd: CGCGCATGCAACTGGCATATAACT, Rev: ATGCTTCCGTTCCCACGTAGACTT
Isg15, Fwd: GGTGTCCGTGACTAACTCCAT, Rev: TGGAAAGGGTAAGACCGTCCT
Ncam, Fwd: CCCAGCCAAGGAGAAATCAG, Rev: TGGCGTTGTAGATGGTGAGG
Musk, Fwd: TACAGAGGGGAGGTGTGTGA, Rev: TCCCGGTAGGAGGTGTTGAA
Chrna1, Fwd: CTCTCGACTGTTCTCCTGCTG, Rev: GTAGACCCACGGTGACTTGTA
Erbb2, Fwd: GAGACAGAGCTAAGGAAGCTGA, Rev: ACGGGGATTTTCACGTTCTCC
Myog, Fwd: GAGACATCCCCCTATTTCTACCA, Rev: GCTCAGTCCGCTCATAGCC
Gadd45a, Fwd: CCGAAAGGATGGACACGGTG, Rev: TTATCGGGGTCTACGTTGAGC
Ctsl, Fwd: GTGGACTGTTCTCACGCTCAAG, Rev: TCCGTCCTTCGCTTCATAGG
4ebp1, Fwd: CACGCTCTTCAGCACCAC, Rev: GGAGGCTCATCGCTGGTAG
Fbxo32, Fwd: GCAAACACTGCCACATTCTCTC, Rev: CTTGAGGGGAAAGTGAGACG
Gabarapl1, Fwd: CATCGTGGAGAAGGCTCTA, Rev: ATACAGCTGGCCCATGGTAG
Nrf2, Fwd: GCAACTCCAGAAGGAACAGG, Rev: AGGCATCTTGTTTGGGAATG
Psma1, Fwd: CATTGGAATCGTTGGTAAAGAC, Rev: GTTCATCGGCTTTTTCTGC
Trim63, Fwd: TGTCTGGAGGTCGTTTCCG, Rev: ATGCCGGTCCATGATCACTT

References

Bolte, S., and F. P. Cordelieres. 2006. 'A guided tour into subcellular colocalization analysis in light microscopy', *J Microsc*, 224: 213-32.

Cuervo, A. M., J. F. Dice, and E. Knecht. 1997. 'A population of rat liver lysosomes responsible for the selective uptake and degradation of cytosolic proteins', *J Biol Chem*, 272: 5606-15.

Supplementary Table 1. Anthropometric parameters of participants of the study

	Young (n=25)	Old (n=99)	p value
**Men/women**	17/8	38/61	
**Age (years) Mean ± SD**	48 ± 7.9	82 ± 8.0	1.24×10^{-38}
**BMI (Kg/m²) Mean ± SD**	30.0 ± 6.6	26.8 ± 4.9	0.017
**Height (cm) Mean ± SD**	166.6 ± 8.8	159.6 ± 11.4	0.013

Supplementary Table 2. Linear regression models of the factors associated with BNIP3 expression.

	Coefficient	95% CI	p value
**Bivariate model (n=121)**			
Age (continuous)	0.012	(-0.008; 0.032)	0.219
Intercept	0.583	(-0.974; 2.139)	0.460
**Multivariate model (n=110)**			
Age (continuous)	0.005	(-0.016; 0.025)	0.658
Gender			
Male	Ref.		
Female	0.546	(-0.090; 1.183)	0.092
BMI (continuous)	0.133	(-0.037; 0.063)	0.599
Intercept	0.440	(-1.721; 2.600)	0.688

Ref: Category of reference; CI: confidence interval

Figure S1. BNIP3 regulates mitophagy and mitochondrial health in skeletal muscle cells.

A) BNIP3 mRNA expression in control (C) and BNIP3 knockdown (BNIP3 KD) C2C12 myotubes (n=10). B) Mitochondrial mass measured by the mitochondrial marker TIM44 protein expression by WB in C and BNIP3 KD cells (n=5). C) Mitochondrial mass measured by mtDNA copy number in C and BNIP3 KD cells (n=5-8). D) Mitochondrial mass measured by Mitotracker Deep Red fluorescence quantification in the absence or presence of 200 nM Bafilomycin A for 16h (n=12). E) Representative immunofluorescence images for COX1 (mitochondrial marker) and LC3 (autophagosome marker) of C and BNIP3 KD myotubes under basal, starvation (EBSS) and bafilomycin A-treated conditions (scale bar 10 μm); F) Representative immunofluorescence images for TOM20 (mitochondrial marker) and LAMP1 (lysosomal marker) of C and BNIP3 KD myotubes (scale bar 10 μm), and quantification of co-distribution between TOM20 and LAMP1 in C and BNIP3 KD myotubes (n=35-40 myotubes). G-H)) Representative WB and quantification of BNIP3, LC3II and PINK1 protein levels in mitochondrial enriched fractions from C and BNIP3 KD myotubes untreated or treated with 1 μM CCCP for 1h (n=4-6). I) Mitochondrial membrane potential measured by TMRM fluorescence quantification in C and BNIP3 KD myotubes (n=9). J) H_2O_2 levels measured by Amplex Red absorbance quantification in C and BNIP3 KD cells (n=5). Data are expressed as mean ± SE. *p<0.05.

Figure S2. BNIP3 regulates lysosomal function in skeletal muscle cells

A) Representative WB and quantification of early (EEA1, Rab5) and late endosomal/lysosomal markers (LAMP1, Rab7) in C and BNIP3 KD myotubes (n=8). B) Representative LAMP1 immunofluorescence image in C and BNIP3 KD myotubes (scale bar 10 μm). C) Representative WB and quantification of cathepsin B (CTSB) and cathepsin D (CTSD) processing in C and BNIP3 KD myotubes (n=6). D) Representative immunofluorescence images of BNIP3, LAMP1 and TOM20 in control C2C12 myoblasts (scale bar 10 μm). E) Quantification of co-distribution of BNIP3 with LAMP1, and BNIP3 with TOM20 in C2C12 myotubes (n=16 myotubes). Data are expressed as mean ± SE. *p<0.05.

Figure S3. BNIP3 repression leads to NFkB-dependent inflammation in muscle.

A) mRNA expression of NFkB target genes in C and BNIP3 KD myotubes (n=12-15). B) mRNA expression of IRF3 target genes in C and BNIP3 KD myotubes (n=12-15). C) BNIP3 mRNA expression in C and BNIP3 KD myotubes (n=6-9). D) H_2O_2 levels measured by Amplex Red absorbance

quantification in C and BNIP3 KD mouse gastrocnemius muscle (n=5). E) Cross-sectional area (CSA) of C and BNIP3 KD gastrocnemius (n=4). Data are expressed as mean ± SE. *p<0.05.

Figure S4. BNIP3 deficiency leads to TLR9-mediated inflammation.

A) H_2O_2 levels in C and BNIP3 KD myotubes untreated or treated with N-acetylcysteine (NAC) 5 mM for 16h (n=5-9). B) Quantification of inflammatory protein expression in C and BNIP3 KD myotubes untreated or treated with NAC (n=5). C) WB analysis of cathepsin B in cytosolic fractions from C and BNIP3 KD myotubes (n=4). CII (SDHA) and LAMP1 were used as mitochondrial and lysosomal markers respectively (present in the pellet) and GAPDH as a cytosolic maker. D) Representative immunofluorescence images for dsDNA and TOM20 in C2C12 myoblasts (scale bar 10 μm). E) Representative immunofluorescence images for dsDNA, TLR9 and LAMP1 in C and BNIP3 KD myotubes (scale bar 10 μm). F) Representative WB of inflammatory markers in C and BNIP3 KD untreated and treated with the TLR9 antagonist ODN2088 (1 μM for 16h). G) mRNA expression of *Nlrp3 and S100a9* inflammatory markers in C cells, and BNIP3 KD cells untreated or treated with TLR9 antagonist ODN2088 (1 μM for 16h) (n=9-12). Data are expressed as mean ± SE. *p<0.05.

Figure S5. BNIP3 repression in old mice enhances muscle atrophy.

A) Quantification of protein expression of NLRP3, p65, ASC and cl-casp1 in gastrocnemius muscle from young (6 months), middle-aged (12 months) and old mice (24 months) (n=4-6). B) Fiber size distribution in gastrocnemius muscle from old C and BNIP3 KD mice (n=6 per group). C) Representative image of hematoxylin and eosin staining in transversal sections of gastrocnemius muscle showing small atrophic fibers (black arrows). D) mRNA expression of several genes associated with denervation in C and BNIP3 KD muscle from old mice (n=5). E) Protein expression of the denervation marker NCAM in C and BNIP3 KD muscle from old mice (n=5). Data are expressed as mean ± SE. *p<0.05.
