# Supplementary material for: Coordination of mitochondrial and lysosomal homeostasis mitigates inflammation and muscle atrophy during aging
Source: Aging Cell. 2022 Mar 9;21(4):e13583. doi: 10.1111/acel.13583 (PMC9009131; doi:10.1111/acel.13583)

Supplementary figure 1

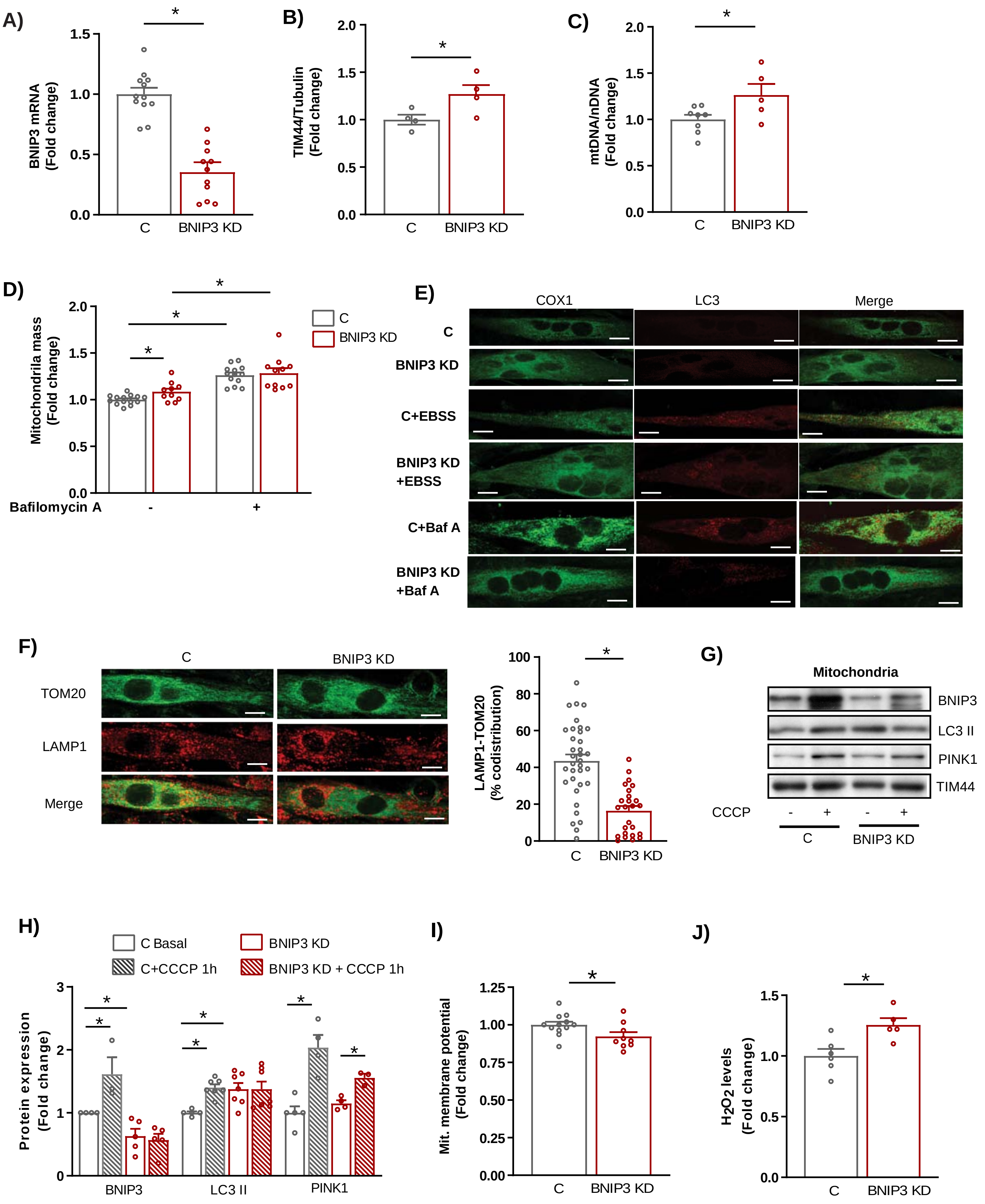

Supplementary Figure 2

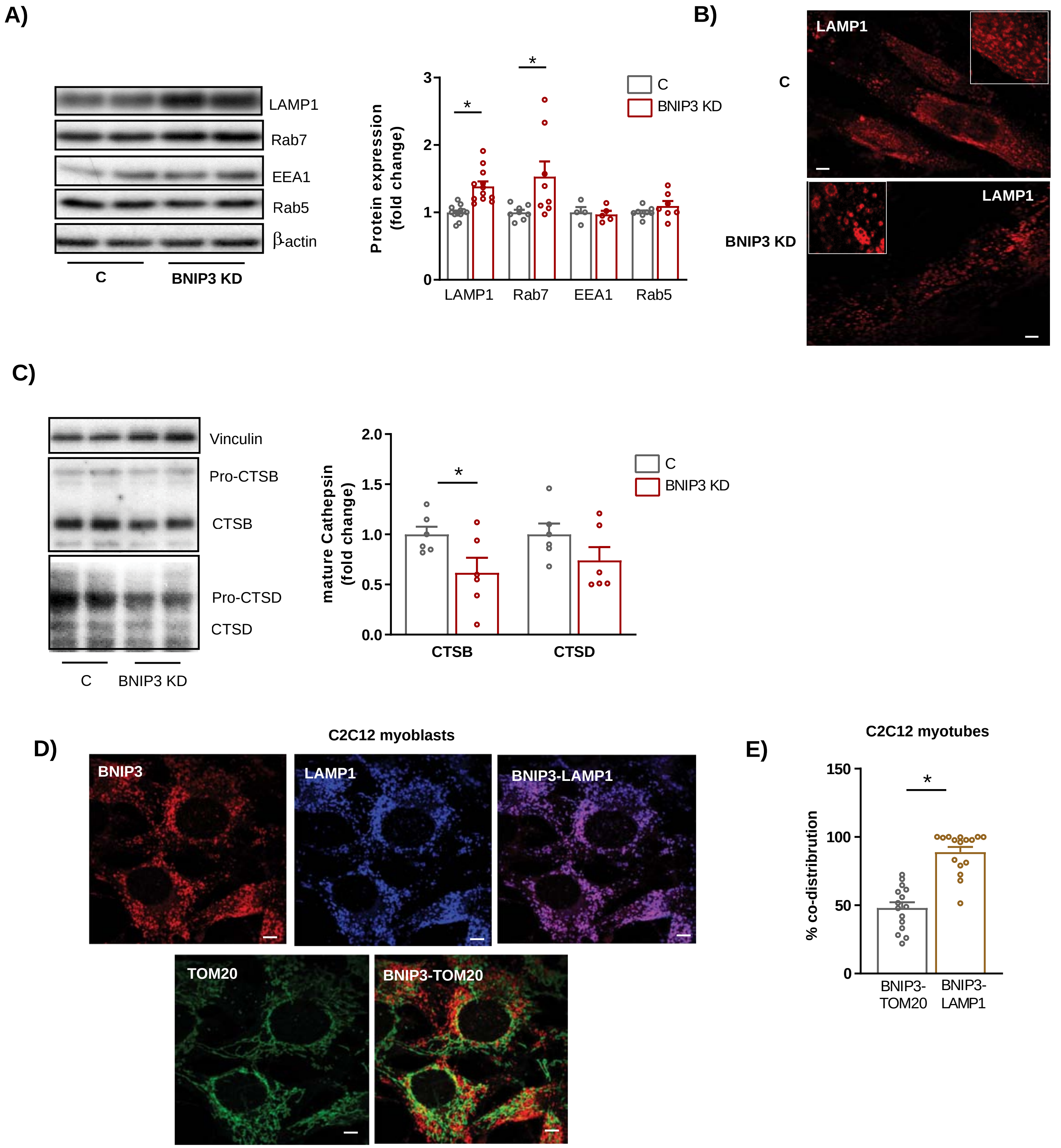

Supplementary Figure 3

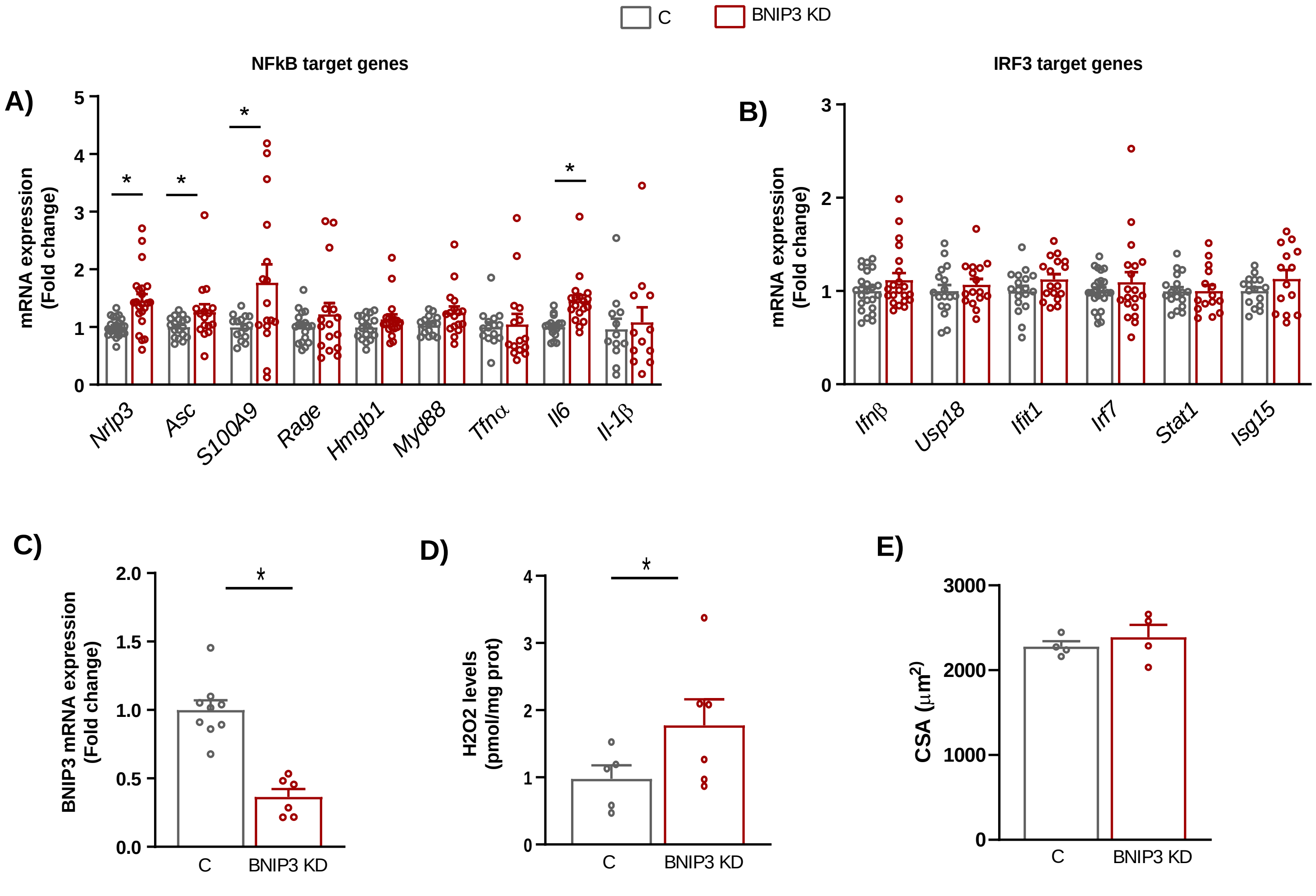

Supplementary Figure 4

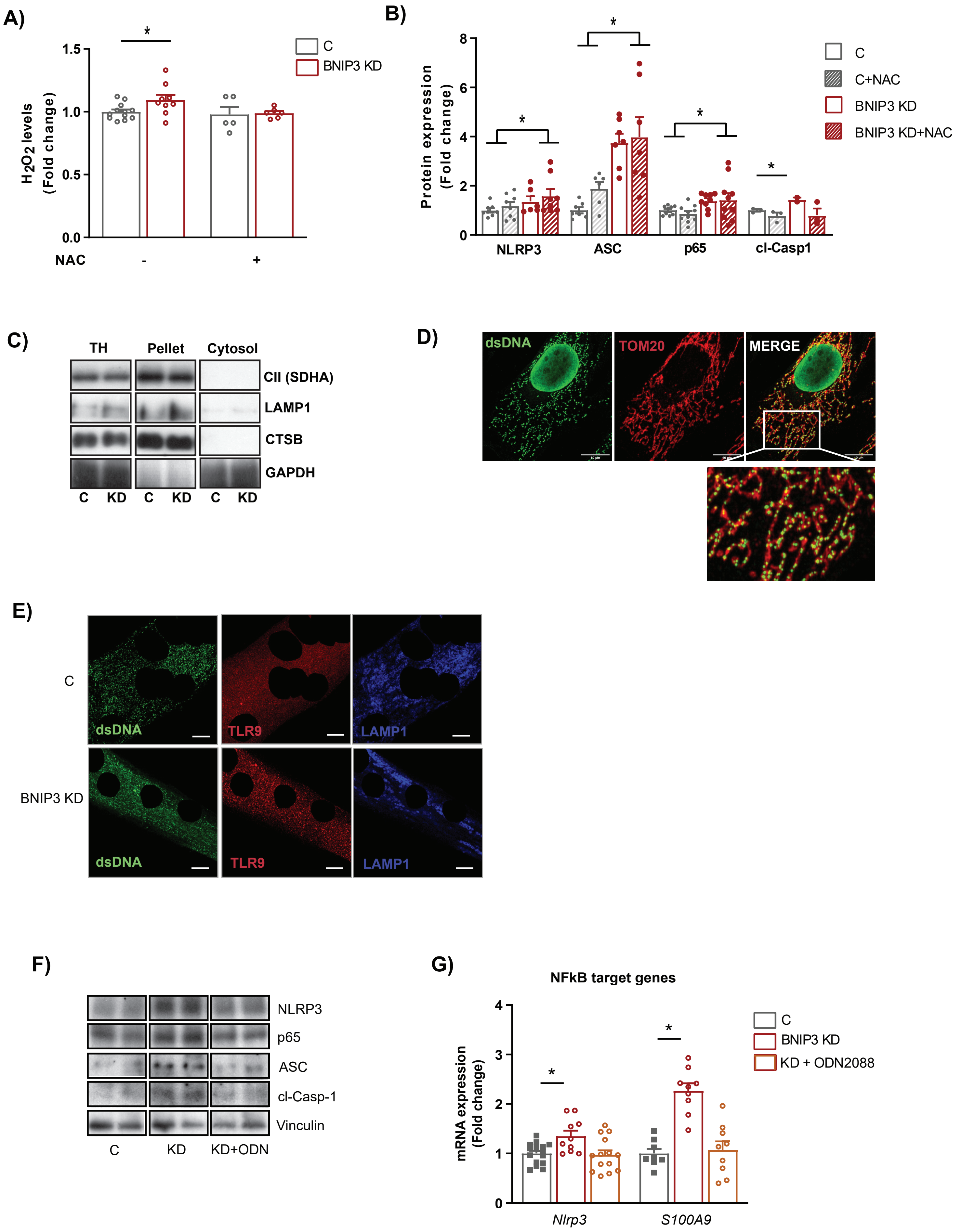

Supplementary Figure 5

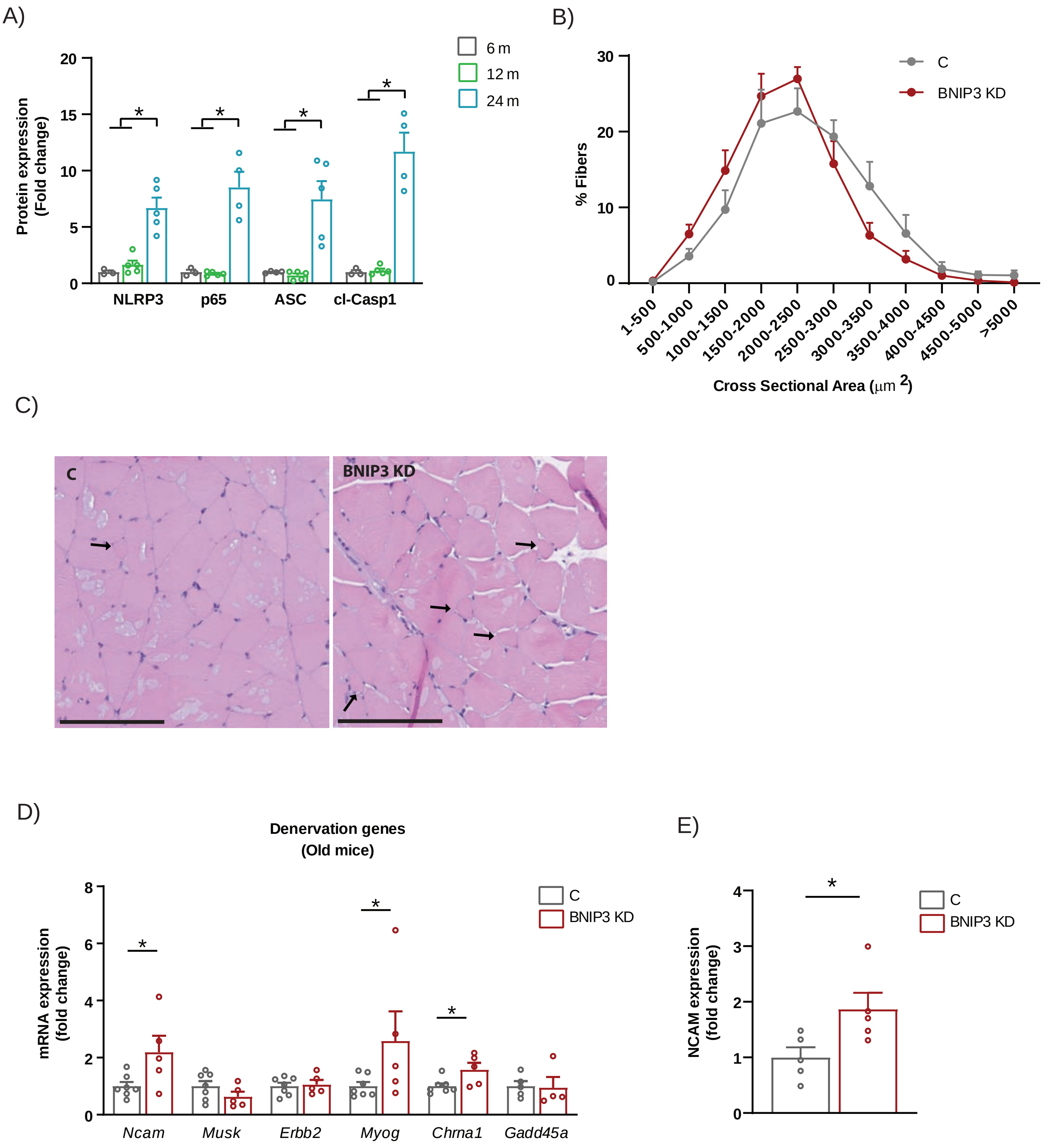

Supplement: Supplementary file 2 — Fig S1‐S5 [file ACEL-21-e13583-s002.pdf]
